# New insights into ocean sunfish (*Mola mola*) abundance and seasonal distribution in the northeast Atlantic

**DOI:** 10.1038/s41598-017-02103-6

**Published:** 2017-05-17

**Authors:** Patricia Breen, Ana Cañadas, Oliver Ó Cadhla, Mick Mackey, Meike Scheidat, Steve C. V. Geelhoed, Emer Rogan, Mark Jessopp

**Affiliations:** 1 School of Biological, Earth and Environmental Sciences, University College Cork, Enterprise Centre, North Mall, Distillery Fields, Cork Ireland; 2 ALNILAM Research and Conservation Ltd, Pradillos 29, 28491 Navacerrada, Madrid Spain; 3 National Parks and Wildlife Service, Department of Arts, Heritage, Regional, Rural and Gaeltacht Affairs, Custom House, Flood Street, Galway, Ireland; 4 Wageningen Marine Research, Haringkade 1, 1976 CP IJmuiden, The Netherlands; 5 Wageningen Marine Research, Ankerpark 27, 1781 AG Den Helder, The Netherlands; 6 MaREI Centre, Environmental Research Institute, University College Cork, Beaufort Building, Haulbowline Road, Ringaskiddy, Cork Ireland

## Abstract

The ocean sunfish, *Mola mola*, is the largest teleost fish in the world. Despite being found in all oceans of the world, little is known about its abundance and factors driving its distribution. In this study we provide the first abundance estimates for sunfish in offshore waters in the northeast Atlantic and the first record of extensive sunfish presence in these waters year-round. Abundance estimates and predictive distributions for sunfish in approximately 300,000 km² of the northeast Atlantic were derived from large scale offshore aerial surveys in 2015–2016 using distance sampling techniques. Generalized additive models of sunfish density were fitted to survey data from 17,360 km of line transect effort resulting in minimum abundance estimates of 12,702 (CI: 9,864-16,357) in the summer (Density = 0.043 ind/km²) and 8,223 individuals (CI: 6,178-10,946) (Density = 0.028 ind/km²) in the winter. Density surface models predicted seasonal shifts in distribution and highlighted the importance of the mixed layer depth, possibly related to thermoregulation following deep foraging dives. The abundance estimate and estimated daily consumption of 2,600 tonnes of jellyfish in the northeast Atlantic highlights the need to re-assess the importance of this species in the pelagic ecosystem, and its role in top-down control of jellyfish blooms.

## Introduction

The Ocean Sunfish (*Mola mola*, hereafter sunfish) is the largest and most fecund teleost fish in the world^1^ and whilst it is considered to be epipelagic, widespread and ubiquitous, little is known about its distribution and habitat preferences^2^. Sunfish are known to be active swimmers showing vertical diving and horizontal migration through the water column^3^. Limited tagging studies on sunfish suggest that they migrate northwards to temperate regions in summer and south to warmer waters in winter^4–6^. Diurnal differences in dive patterns have also been detected, with deeper dives occurring during daylight hours^3, 7^.

In the northeast Atlantic, sunfish are known to feed largely on low nutritional value jellyfish species including *Rhizostoma octopus*, *Chryasora hysoscella* and *Cyanea capillata*, and are frequently sighted near large jellyfish aggregations^8^. While recent studies suggest a more cosmopolitan diet, particularly for juveniles and sub-adults^9^, jellyfish predators such as sunfish are likely to be key ecosystem species with an important role in controlling large jellyfish blooms^1^. A better knowledge of their distribution, abundance and life history is therefore critical in understanding their trophic position and functional role in the marine ecosystem. Furthermore, describing and understanding the relationship between species distribution and environmental variables is critical for effective biological conservation and management over a large spatial scale.

Accurately describing spatial trends in distribution and generating estimates of abundance of highly mobile marine predators that may spend little time at the surface, such as sunfish, are difficult tasks. For cetaceans and some shark species, distance sampling^10^ from ship-based and aerial surveys has been used to generate estimates of abundance^11–13^. Sunfish records are notably sparse, particularly from ship-based surveys probably due to the negative reaction of sunfish to the vessel’s presence. Furthermore, previous aerial survey effort in the northeast Atlantic has been restricted to summer months^14^ resulting in significant gaps in data collection during other seasons.

Due to the likely role sunfish play in regulating jellyfish blooms^1^, and the recent updating of the species to ‘vulnerable’ by the International Union for the Conservation of Nature (IUCN) because of localised declines and high bycatch in parts of its range^15^, we aimed to determine the abundance and distribution of this large pelagic predator in offshore waters of the northeast Atlantic. Abundance estimates are critically important for ecosystem food web models to examine trophic interactions and the effects of large-scale perturbations such as those predicted under various climate change scenarios, while information on distribution is also particularly relevant for marine spatial planning and effective conservation.

## Results

**Figure 1:**
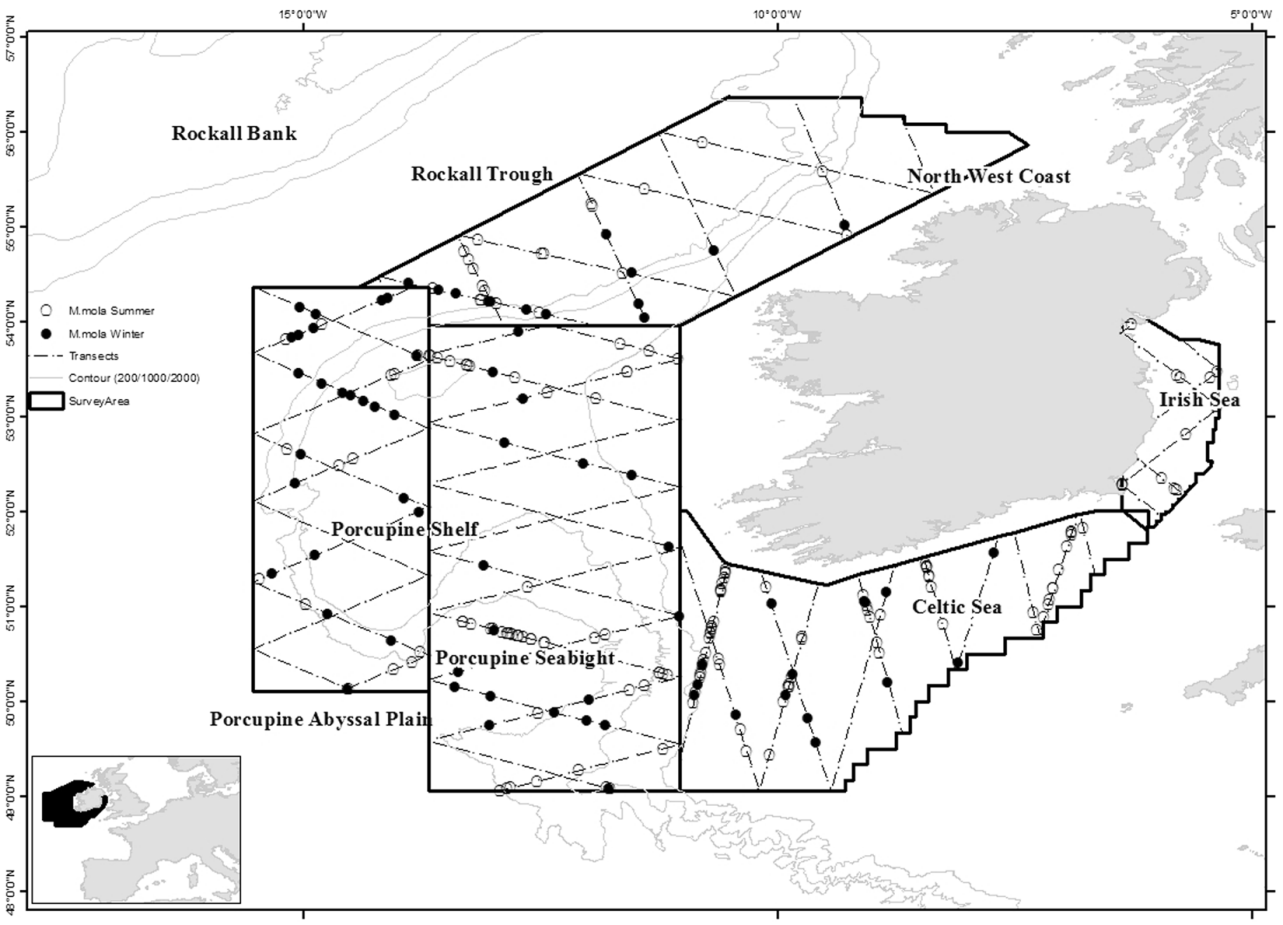
Study area within the northeast Atlantic Ocean showing key areas discussed in the text. Dashed zigzag lines show the transects flown twice within each survey stratum, once per season. Closed dots show winter sightings and open circles show summer sightings. Map generated using ArcGIS 10.2. http://www.esri.com/.

Sunfish were sighted by the aerial team during both summer and winter surveys, indicating a year round presence at these latitudes (Fig. 1). They were recorded in all survey areas in the summer, but records were absent from the Irish Sea in winter. There were 172 sightings consisting of 181 individuals recorded in summer and 72 sightings of single individuals recorded in winter (Table 1). Sightings were mostly of individual animals but occasionally groups of up to four sunfish were sighted during the summer months.

**Table 1:** Design-based abundance and density estimate results for sunfish with confidence intervals and coefficients of variation (CV) in each case.

Season	#Sightings/#Individuals	#Sightings used	Model type (Hazard rate/Half-normal)	Abundance	Density (ind/km²)	Upper confidence interval	Lower confidence interval	CV (%)
Summer	172/181	149	HR	12,702	0.043	9864	16,357	12
Winter	72/72	67	HN	8485	0.029	6214	11,586	15
Combined	237/253	216	HN	10,744	0.036	8882	12,997	9

### Design-based method

For the CDS model in the summer a hazard-rate model with no adjustment terms was chosen as the best model, this provided an *esw* of 226 m and a minimum abundance estimate of 12,702 animals (CI: 9864-16,357; CV = 0.12) (Table 1). For the winter data a half-normal model was used with an *esw* of 147 m and an abundance estimate of 8223 (CI: 6178-10,946; CV = 0.15) (Table 1). Combining the seasons sightings, a half-normal model was used with no adjustment terms which gave an *esw* of 181 m and abundance estimate of 11,288 (CI: 8867-14,370; CV = 0.09) (Table 1).

### Model-based method

**Figure 2:**
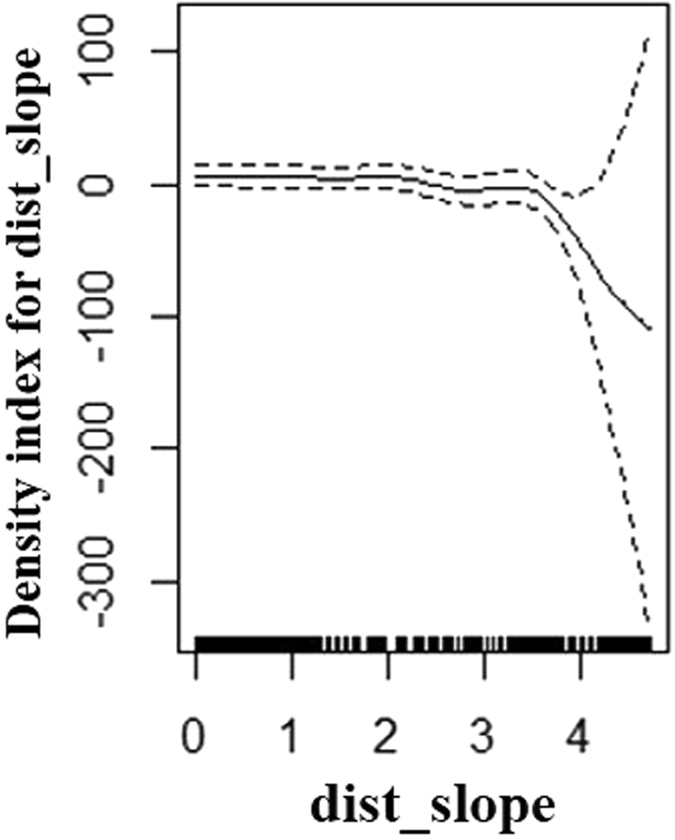
Shape of the functional form of the smoothed covariate (distance to slope) in the winter model. A zero on the Y axis corresponds to no effect of the covariate on the response variable (individual sightings). The dashed lines represent twice the standard error of the estimated curves (95% confidence bands). The locations of the observations are plotted as small ticks along the X axis. Figure generated using R version 3.3.1 https://www.r-project.org/.

**Figure 3:**
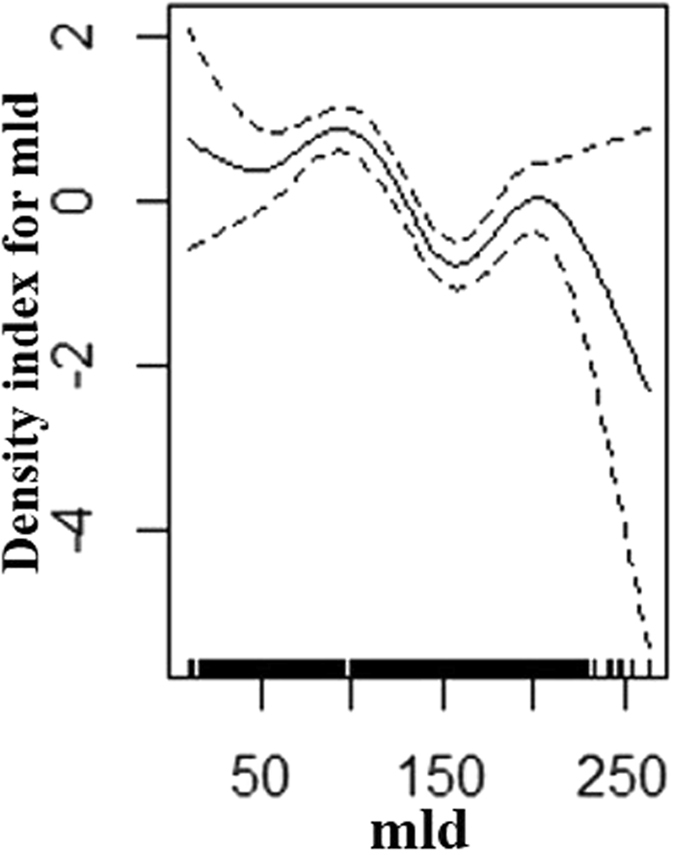
Shape of the functional form of the smoothed covariate (mixed layer depth) in the combined model. A zero on the Y axis corresponds to no effect of the covariate on the response variable (individual sightings). The dashed lines represent twice the standard error of the estimated curves (95% confidence bands). The locations of the observations are plotted as small ticks along the X axis. Figure generated using R version 3.3.1 https://www.r-project.org/.

For summer the best model had a Tweedie distribution with a log link function. It included an interaction term between latitude and longitude which was significant (p << 0.001), the total deviance explained was 13.6% (Table 2). In winter, the best model also had a Tweedie distribution with a log link function. It also included latitude and longitude as a significant interaction term (p << 0.001) as well as the ‘distance to the slope’ parameter (p < 0.001) (Fig. 2). The overall deviance explained was 16% (Table 2). For the combined data the best model (Distribution: Tweedie, Link function: log) included the latitude, longitude interaction (p << 0.001) and mixed layer depth (p << 0.001) (Fig. 3) with 9.9% deviance explained (Table 2).

**Table 2:** Density surface modelling results with the environmental covariates best predicting sunfish density across the study area.

Season	Covariates	edf	P value	Deviance explained (%)
Summer	Latitude*Longitude	27.6	<0.001	13.6
Winter	Latitude*Longitude	27.7	<0.001	16.0
	Distance to Slope	1.0	<0.004	
Combined	Latitude*Longitude	23.3	<0.002	9.9
	Mixed layer depth	4.8	<0.003

Comparing abundance and density estimates from the two methods shows very similar results. CV values are higher for the model-based approach compared to the design-based approach, therefore we have reported the abundance estimates with the lowest CVs which are more precise.

**Figure 4:**
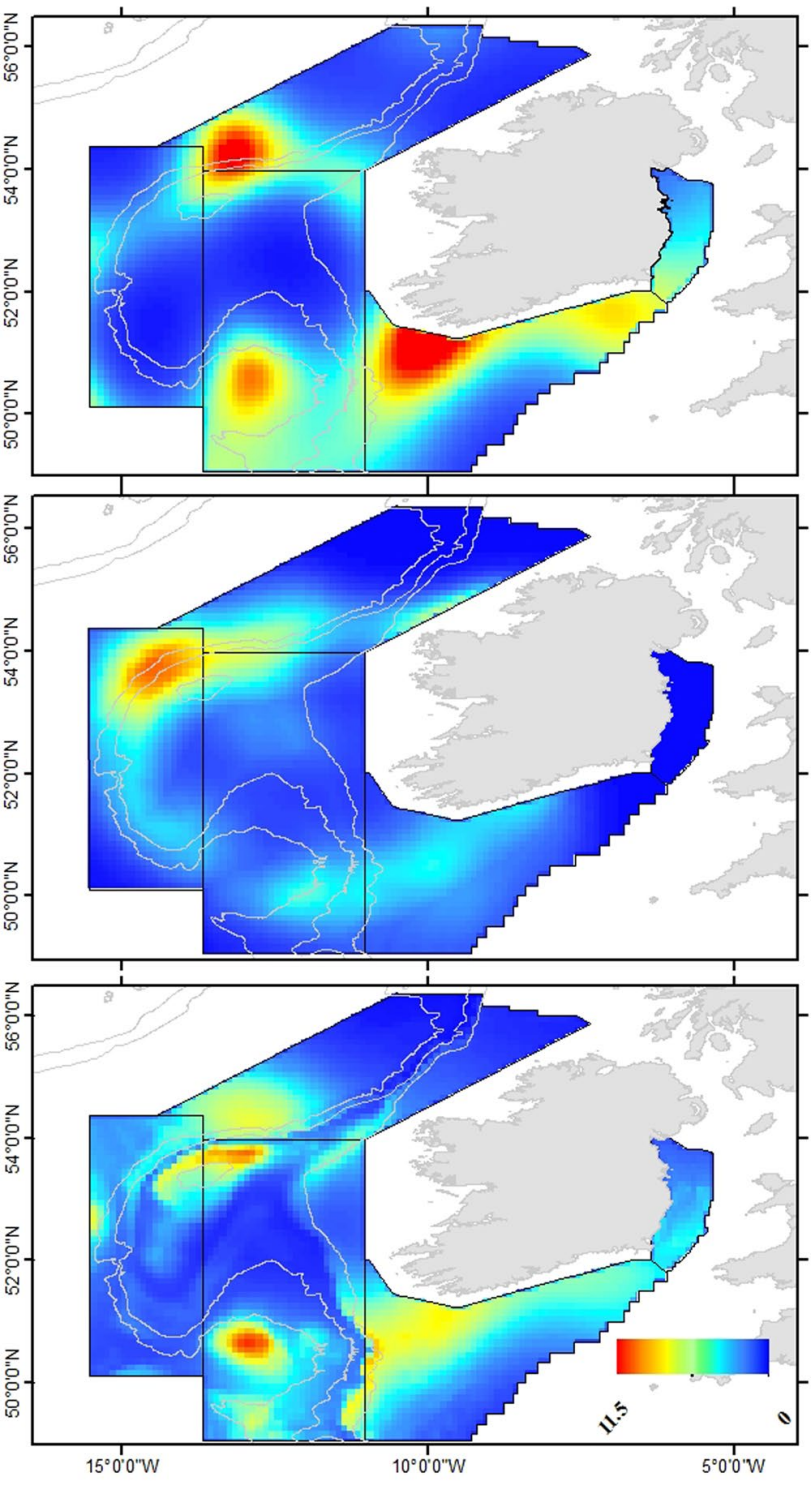
Density surface models for (**a**) summer survey data (**b**) winter survey data and (**c**) combined summer and winter data. Red colours indicate high density, dark blue colours indicate low density. Map generated using ArcGIS 10.2. http://www.esri.com/.

Predictive distribution across the survey area showed the Celtic Sea area to have a comparatively high density of sunfish during the summer months (Fig. 4). The Irish Sea showed presence in the summer, however no sunfish were sighted there in the winter (Figs 1 and 4). The Porcupine Seabight was recorded as a key area of higher density, particularly in the summer, which was also reflected in the combined (summer and winter) model. An area of higher density was also predicted by the model to occur at the northern reaches of the Porcupine Shelf and adjacent continental slope in both survey seasons. Interestingly, in all three distribution models there was a distinct area of lower sunfish density indicated between latitudes 51°N and 54°N in waters overlying the continental shelf and northern reaches of the Porcupine Seabight (Fig. 4).

## Discussion

Our study area covers a vast off shore region, some 300,000 km^2^ in extent, spanning large parts of the Irish and Celtic Seas as well as the deeper waters of the Atlantic to the west of Ireland. To our knowledge this is the first study to estimate abundance of sunfish at such a scale and, by incorporating habitat variables into distribution models, we provide the first abundance and density estimates for sunfish in the northeast Atlantic Ocean. Abundance is higher in summer than winter, and density estimates indicate that there may be twice as many individuals present in the region in summer compared to winter (i.e. 0.028 ind/km² in winter compared to 0.043 ind/km² in summer). This value is lower, but similar to the 0.09 ind/km^2^ reported in Houghton *et al*.^14^. We surveyed over a broader offshore scale, encompassing a wider range of marine and oceanographic environments and thereby potentially incurring greater variability in sunfish presence/absence than might be the case in a shallower coastal ecosystem.

Current literature describes sunfish as a migratory species in Atlantic waters. Off the coast of North America it is present in the Gulf of Maine in summer, whilst moving south in late summer and early autumn and vacating the Gulf of Maine by October. Migrating animals moved as far south as the Bahamas and the Gulf of Mexico^5^. In the eastern Atlantic it moves northeast during the summer to feed on jellyfish aggregations and then moves southwards to warmer waters during the winter time^16^. Seasonal inshore migrations around the English Channel have also been reported, which are thought to coincide with invasions of medusa, salps and ctenophores, key food sources for sunfish^2^. Results from our surveys show that some sunfish are present in the waters of the northeast Atlantic year-round, and the seasonal differences in abundance provide evidence for some seasonal movement in line with recent tagging studies^17^. Seasonal movement may be to more southerly waters or inshore waters which were not covered in the current study, or sunfish may spend more time at depth in the winter months. Invasions of gelatinous zooplankton are more common in summer than winter^18^ and sunfish are considered absent inshore in this region during winter^2^. The results presented here indicate the absence of sunfish in the shallow coastal waters of the Irish Sea in winter so it is more probable that sunfish are moving south in the winter months. Although sunfish size was not systematically recorded, observers noted that sunfish less than 1 m in length, i.e. most likely juveniles or sub-adults^1^ were sighted year round whilst large individuals up to 3 m in length were recorded on several occasions during the summer, but never in winter. It has been suggested that juveniles and adults forage on different food resources, with adult diet made up mostly of gelatinous zooplankton whilst juveniles are mostly benthic feeders^9, 19^. It is possible that the seasonal differences in abundances and distribution are therefore due to adult migration to find improved jellyfish foraging whilst the less specialist juveniles remain in the region all year round.

We were not able to generate correction factors for availability bias which is likely to be significant for sunfish since, they do not rely on surfacing to breathe. Variation in availability bias is species-specific, and in the case of sunfish may also vary depending on other factors such as temperature, depth, age/size and prey availability. A previous study by Houghton *et al*.^8^ cited evidence from Cartamil & Lowe^3^ that indicated sunfish spend between 20–30% of their time in the top 5 m of the water column, prompting them to predict that there is approximately a 1 in 4 chance of observing sunfish at the surface. Similar figures are reported elsewhere in the literature^7, 16, 17, 20^. However, these studies tended to restrict their predictions to the top 10 m of water, beyond what is visible from an aircraft. Without detailed information on the percentage of time spent at the surface (<5 m) in this region, it is not currently possible to generate a robust correction factor that addresses the availability parameter. However, if we were to consider estimates of 20–30% of time in surface waters as representative for juvenile and adult sunfish in our region, the number of sunfish present in our study area could be up to five times greater than the estimates presented here. This suggests that sunfish are much more abundant in the northeast Atlantic than previously thought.

There is an active commercial fishery for sunfish in Japan and Taiwan^1, 21^ and, while not directly targeted elsewhere in the world, sunfish are known to be bycaught in large numbers. The largest threat from bycatch is likely to occur in drift net fisheries, particularly in the Mediterranean Sea, where as much as 93% (1737 individuals) of the catch by number can consist of sunfish bycatch^21^. High bycatch figures have also been recorded in the Californian swordfish (*Xiphias gladius*) fishery (comprising 29% of the recorded catch) outnumbering the target species^3^, and in the Cape horse mackerel (*Trachurus capensis*) midwater trawl fishery off South Africa where it can make up to 51% of all bycatch^22^. There is also evidence that a high bycatch of sunfish occurred in the northeast Atlantic from a surface driftnet fishery targeting albacore tuna (*Thunnus alalunga*)^23^.

Our predictive GAM models indicate several areas of high sunfish density within our study area. The Celtic Sea area has particularly high density in summer months, consistent with other species with a northward summer migration such as leatherback turtles^8^ and basking sharks ^24^. ‘Distance to slope’ is a significant variable in the predictive GAM for winter, and models predict high density just off the continental shelf characterised by a steep gradient where seafloor depth drops sharply from 200 m to a deep trough area over 2000m in depth. The joining of deep oceanic and continental shelf waters in this area in combination with the North Atlantic Current and prevailing southwesterly winds create productive waters for sunfish foraging. Although temperature is thought to be significant in sunfish distribution/migration, temperature was not retained in any of the distribution models explored during the present study. Nakamura *et al*.^25^ suggest that sunfish spend time in surface waters in order to rewarm following deep dives. Their tagged sunfish experienced ambient temperatures of 16–22 °C at the surface, and <10 °C at depths over 100 m. In our study, sunfish were recorded in sea surface temperatures ranging from 12.3–15.8 °C in summer, consistent with other studies in the region^17^ and 8.6–11.9 °C in winter, the minimum value of which is below the thermal preference reported in other studies^2, 25^. This represents an average difference of 6.1 °C between surface and bottom waters in summer and 2.5 °C in winter. The mixed layer depth represents the boundary between the warmer surface waters and colder waters at depth and has previously been suggested as an important factor in the foraging ecology of sunfish, with Cartamil & Lowe^3^ suggesting that sunfish return to the mixed layer after dives to depths of up to 600 m. In the present study the mixed layer depth was retained as a significant explanatory factor in our distribution models with the results showing a preference by sunfish for shallower mixed layer depths, corresponding to higher sunfish density in the Celtic Sea and, in certain areas, deeper waters just off the continental shelf. The temperature difference between surface and bottom temperatures, and retention of mixed layer depth in our models may suggest that a return to the mixed layer depth also aids thermoregulation for repeat dives during daytime although the role of the mixed layer in thermoregulation is yet to be proven^7^. Lower statistical power, indicated by the low deviance explained in the models, combined with high significance towards the latitude and longitude interaction in the seasonal models could be masking the importance of the mixed layer depth variable in the seasonal models. Increased effort via future aerial surveys of the kind performed here would help to shed further light on the importance the mixed layer depth plays in the horizontal and vertical distribution of sunfish.

The main prey of adult sunfish, ‘gelatinous’ jellyfish, is a key element of the northeast Atlantic ecosystem. Jellyfish blooms have increased over the last 10 years^18^, with numbers also increasing in winter months^26^ attributed to climate change^27, 28^. Large jellyfish blooms can cause serious economic losses by negatively affecting fish recruitment^26^ and stock size^18^, by causing fish kills in aquaculture^29^, the closure of beaches^30^ and power outages following the blockage of cooling water intakes at coastal power plants^31^. Gibbons & Richardson^18^ suggest that due to the scarcity of obligate jellyfish feeders such as turtles and birds, they are unlikely to exert top-down control on the numbers of jellyfish. However, our results clearly suggest that sunfish are much more abundant that previously thought, and are likely to play a key role in the control of jellyfish numbers in the northeast Atlantic.

Nakamura *et al*.^25^ deployed accelerometers and animal-borne cameras on sunfish (1.05–1.91 m total length) in the Pacific Ocean. They visually identified feeding events on gelatinous zooplankton (siphonophores, scyphozoa and ctenophores) in camera data and extracted the movement characteristics of feeding events from corresponding accelerometry data to detect 5,131 feeding events 7 sunfish over 4–6 day deployment periods. This roughly equates to an average of 147 prey captures per sunfish per day. Given the similar size range of sunfish tagged in the Pacific and noted in our surveys, assuming a similar prey capture rate, and wet mass of jellyfish prey in our survey region, we can produce a gross estimate of jellyfish consumption by sunfish across our study area. Average wet mass across all scyphozoan jellyfish sampled by Doyle *et al*.^32^ over summer months in the northeast Atlantic was 1.45 kg. Multiplying this by an average of 147 prey captures per sunfish per day, gives an estimate of approximately 213 kg wet mass of jellyfish consumed per day. A similar approach was taken by Heaslip *et al*.^33^ who recorded 601 jellyfish prey captures by an obligate jellyfish predator, the leatherback sea turtle (carapace length 143–162 cm, estimated body mass 455 kg), using animal-borne video cameras. They used prey capture rates and average prey sizes to estimate jellyfish consumption by leatherback turtles of 330 kg per day equating to approximately 73% of body mass per day^33^, beside which, our estimate compares well. Leatherbacks have been shown to migrate out of the northeast Atlantic during winter^34^, the estimated abundance and continuous presence of sunfish found in the present study suggests that sunfish have a broader thermal range compared to leatherbacks and highlights the need to re-assess the importance of this species in the pelagic ecosystem. At even a very conservative estimate of sunfish abundance derived in this study, sunfish in the study area alone maybe consuming upwards of 2,600 tonnes of jellyfish prey per day in summer. Few marine ecosystem models currently include jellyfish and therefore jellyfish predators, or the trophic role they play in the ecosystem^35^. Ecosystem models focusing explicitly on these pelagic components of the marine ecosystem will help to answer questions regarding the role of sunfish in top-down control of jellyfish populations, as well as exploring the relationship between sunfish bycatch mortality and the occurrence of jellyfish blooms.

## Methods

### Survey design and operation

In 2015 and early 2016, aerial line transect surveys totalling 17,360 km in length were conducted over a total survey area of 299,247 km² in order to estimate the abundance and distribution of marine vertebrates within offshore waters of the Irish EEZ in the northeast Atlantic (Fig. 1). The survey area was divided into four contiguous blocks (i.e., strata). Within each stratum two survey transects, consisting of multiple zigzag lines, were generated randomly to allow equal coverage probability^10^ and minimise design-based bias in the spatial distribution of aerial transect coverage. The same tracklines were flown in both summer and winter. Summer surveys were flown in June/July 2015 and winter surveys took place between November 2015 and February 2016. Surveys were flown in a high-wing, twin-engine Britten Norman Islander fitted with bubble windows to provide observers with unrestricted views of the area underneath and abeam of the aircraft. Surveys were flown at a height of 183 m and a speed of 90 knots. Two trained visual observers with extensive previous survey experience were seated on either side of the plane and they continuously observed the sea surface below the aircraft out to 500 m whilst two further individuals acted as data loggers. All megafauna including marine mammals, seabirds, sharks, turtles and sunfish were recorded by the survey team. Data recorded included GPS location, date and time of sighting, declination angle to the animal when abeam, group size and observer name. Weather conditions including Beaufort sea state, cloud cover, glare and subjective observation conditions were recorded at the beginning and end of each transect line and any time during survey effort when the observer considered that observation conditions had changed. Surveys were flown in Beaufort sea state 3 or less where possible. The perpendicular distance from the trackline to the sighting or centre of the recorded group was calculated trigonometrically using the declination angle and height of the plane.

### Data analysis

Two methods were used to estimate sunfish abundance within the survey area: 1. A design-based approach using equal coverage probability and distance sampling techniques^10^ and 2. A model-based method which combines the first approach with statistical modelling using environmental covariates to spatially model the distribution and abundance of sunfish throughout the survey area^36, 37^.

A matrix of grid squares measuring approximately 0.1 × 0.1 decimal degrees was generated across the study area, and transects flown were mapped and divided into 1569 segments by clipping them to the grid squares of the survey area to ensure homogenous effort type in individual grid cells (Mean: 5.4 km, Min: 0.001, Max: 29.26 km). For model-based methods, environmental covariates were related to individual grid cells (for spatial predictions) and to individual segments (for spatial modelling).

### Design-based method

Data were analysed using DISTANCE 6.2^10^ software using the conventional distance sampling (CDS) method. In CDS, animal abundance in each stratum is estimated by:

1 $$\hat{N}=A\,\frac{n}{2L\hat{{\rm{\mu }}}}\hat{E}\,\lfloor s\rfloor $$

where, for each stratum, A is the area (km²), L is the total search effort (km), n is the number of sightings, $\hat{{\rm{\mu }}}$ is the estimated effective strip half-width (*esw*) and $\hat{{\rm{E}}}[{\rm{s}}]1/2$ is the estimate of mean group size. A variance estimate for $\hat{{\rm{N}}}$ is obtained by combining the variance estimates of the three components; encounter rate, detection function and group size, using the delta method^10^. Both half-normal and hazard rate models were tested in model development. The minimum value of the Akaike Information Criterion (AIC), along with the performance of the qq plot, and goodness of fit tests (Cramer von Misee, Chi squared, Kolmogorov-Smirnov) were used to choose between models. Multi-covariate Distance Sampling (MCDS) analysis, where covariates which affect the observers ability to detect the animal (e.g. seastate, cloud cover, glare etc) are included in the estimation process, was also attempted but in all cases the CDS detection functions and abundance estimates for sunfish were found to be most suitable. Models were generated for both summer (2015) and winter (2015/2016) separately, and on the combined summer and winter datasets.

### Density Surface Modelling

Using the detection function generated in the design-based method, abundance was estimated for individual segments and the effective strip half-width (*esw*) was calculated. Generalised Additive Models (GAMs) were then used in statistical software ‘R’^38^ (‘*mgcv’* package) to model abundance related to environmental co-variates. This method can increase the precision of abundance estimates^13^ and generate density maps for the study area which can be used for further spatial analysis. Environmental covariates covering 7622 grid squares of 0.1 × 0.1 decimal degrees (dd) across the entire survey area were used in density surface modelling. Topographic variables included seafloor depth, slope, distance to the continental slope and distance to the coastline. Oceanographic variables included sea surface temperature, sea bottom temperature, mixed layer depth and chlorophyll-a concentration. Depth was downloaded from the GEBCO 2014 dataset in 30 sec arc grid. It was aggregated to 0.1dd scale using the mean value of each cell. The depth layer was then used to generate the slope layer using the ‘SLOPE’ tool in ArcGIS 10.2 and a ‘distance to slope’ layer was also generated using measure tools in ArcGIS as the distance to the edge of the continental shelf. Sea surface temperature (sst), sea bottom temperature (sbt) and mixed layer depth (mld) were downloaded as monthly modelled means on the 15^th^ of each month from the Marine Institute Data Portal (http://data.marine.ie/). Resolution was available as 0.025dd grid squares and scaled up to 0.1dd. Chlorophyll *a* (mg/m^3^) was used as a proxy measure of primary productivity and was available from the NEO NASA website as a floating point GeoTIFF. The best resolution available for this layer was 0.1dd. Finally, a ‘distance from coast’ layer was generated from the survey area grid squares and a European boundary file using the MEASURE tool in ArcGIS 10.2.

Models were generated for individual sightings for Summer 2015, Winter 2015/16 and both seasons together using the model structure:

2 $${\hat{{\rm{N}}}}_{{\rm{i}}}=\exp [\mathrm{ln}({{\rm{a}}}_{{\rm{i}}})+{{\rm{\theta }}}_{0}+\sum _{{\rm{k}}}{{\rm{f}}}_{{\rm{k}}}({{\rm{z}}}_{{\rm{ik}}})]$$

where *a*_*i*_ is the effective search area for the *i*th segment (length of the segment multiplies by twice the *esw*), θ_o_ is the intercept, *f*_*k*_ are smoothed functions of the explanatory variables and *z*_*ik*_ is the value of the *k*th variable in the *i*th segment.

Models were fitted using a forward stepwise selection procedure whereby variables were individually added to the model if the overall fit was improved by doing so. This was continued iteratively until the fit could not be improved further. Models with the best fit were selected using the lowest Generalised Cross Validation score (GCV), the percentage of deviance explained and the probability that the variable was included in the model by chance.

Models were not corrected for perception bias (the ability of the observer to see everything on the trackline when it is available to be detected), however using trained and experienced observers minimises perception bias^13^. Availability bias (the availability of sunfish for counting on the trackline) was also not accounted for as there is little published information on how much time sunfish spend at the sea surface in the northeast Atlantic. Furthermore, differences between seasons, individual size and ontogenetic differences in diet^9^ likely influence time at the surface for this species. Our results should therefore be considered as minimum abundance estimates.
